# Pregnant women’s knowledge, attitude, and practice towards COVID-19 infection prevention in Ethiopia: A systematic review and meta-analysis

**DOI:** 10.1371/journal.pone.0276692

**Published:** 2022-10-26

**Authors:** Ayenew Mose, Amare Zewdie, Tadesse Sahle

**Affiliations:** 1 Department of Midwifery, College of Medicine and Health Science, Wolkite University, Wolkite, Ethiopia; 2 Department of Public Health, College of Medicine and Health Science, Wolkite University, Wolkite, Ethiopia; 3 Department of Nursing, College of Medicine and Health Science, Wolkite University, Wolkite, Ethiopia; Federal University of Ceara, UNITED STATES

## Abstract

**Background:**

Coronavirus disease (COVID-19) infection during pregnancy causes adverse maternal and perinatal outcomes such as preterm birth, low birth weight, severe illness, intensive care unit admission, mechanical ventilation, and death. Pregnant women’s knowledge, attitude, and practice (KAP) towards COVID-19 infection prevention are crucial to ensure the health of the mother and foetus. Therefore, this systematic review and meta-analysis aimed to estimate the pooled prevalence of pregnant women’s KAP towards COVID-19 infection prevention in Ethiopia.

**Methods:**

We searched PubMed, Scopus, Google Scholar, African Online Journal, and Web of Sciences database to retrieve related articles. Preferred Reporting Items for Systematic Review and Meta-Analysis (PRISMA) guideline was used. Funnel plot and Eggers test were done to assess publication bias. Cochrane Q-test and I^2^ statistic were done to chick evidence of heterogeneity. Subgroup analysis was computed based on the study region and year of publication. Data were extracted using a Microsoft Excel spreadsheet and analyzed using STATA version 14 statistical software. Weighted inverse variance random effect model was run to estimate the pooled prevalence of pregnant women’s KAP towards COVID-19 infection prevention.

**Results:**

A total of 9 studies with 4,103 pregnant women were included. The pooled prevalence of knowledge, attitude, and practice towards COVID-19 infection prevention among pregnant women’s in Ethiopia were 60.24% (95% CI; 53.69 to 66.79, I^2^ = 95%), 62.46% (95% CI; 45.68, 79.23, I^2^ = 98.8%), and 52.29% (95% CI; 43.91%-60.66% I^2^ = 96.5%) respectively. Maternal age (AOR = 1.87, 1.40–2.49), residence (AOR = 2.23, 1.50–3.31), secondary and above educational status (AOR = 3.36, 2.46–4.58), good knowledge (AOR = 2.73, 2.18–3.41), and fear of COVID-19 infection (AOR = 2.60, 1.78, 3.80) were factors associated with COVID-19 infection prevention practice among pregnant women’s in Ethiopia.

**Conclusion:**

The knowledge, attitude, and practice of COVID-19 infection prevention among pregnant women were low. Therefore, policymakers, maternal and child health program planners, and stakeholders should target to improve pregnant women’s awareness regarding COVID-19 infection preventive measures.

## Background

Coronavirus disease 2019 (COVID-19) is an infectious disease caused by severe acute respiratory syndrome coronavirus-2 (SARS-CoV-2) virus which was started in Wuhan, China [1]. As of 26 July 2022, it has resulted in 568,773,510 confirmed cases of COVID-19 and 6,381,643 deaths globally. Similarly, in the African continent, 9,192,139 confirmed cases and 173,946 deaths were reported [2]. In Ethiopia, as of 26 July 2022, there have been 491,759 confirmed cases of COVID-19 with 7,566 deaths reported [3].

As of 1 March 2021, more than 73,600 infections and 80 maternal deaths were reported among infected pregnant women in the United States [4]. As of 6 October 2021, a total of 1,637 SARS-CoV-2 infections during pregnancy and 15 deaths were reported in Mississippi [5]. Immunologic and physiologic changes occurred during pregnancy predispose women to develop severe illness from respiratory infections including SARS-CoV2 and respiratory syncytial virus influenza virus (RSV) [6, 7].

The World Health Organization has recommended several COVID-19 preventive measures such as hand washing, wearing a face mask, and maintaining physical distance to halt the spread of the disease and its associated morbidity and mortality [8]. Since the beginning of the pandemic, Ethiopia has implemented several COVID-19 mitigation measures [9].

Evidence shows that those pregnant women who had been infected with the COVID-19 virus have developed adverse maternal and perinatal outcomes such as preterm birth, low birth weight, severe illness, intensive care unit admission, mechanical ventilation, and death [10–12]. Pregnant women and their foetus are the vulnerable group of the population and are susceptible to COVID-19 infection [13]. This is due to an increased risk of hospital admission, altered immune response, and intensive care unit stay compared with their nonpregnant counterparts [14].

A variety of findings were reported regarding pregnant women’s (KAP) towards COVID-19 infection prevention. For instance, the KAP of COVID-19 infection prevention in India, Northern Ghana, and South Africa were 46.6% to 92.7% [15–17]. In Ethiopia, pregnant women’s COVID-19 infection prevention practice was reported in the Oromia region 43.6%, in the Southern region 76.2%, and in the Amhara region 44.8% [18–20]. Maternal age, educational status, and residence were some of the factors associated with COVID-19 infection prevention practice among pregnant women’s in Ethiopia [21, 22].

While several studies were published in Ethiopia, there were inconsistencies of findings regarding the pregnant women’s KAP towards COVID-19 infection prevention. In conclusion, understanding the overall KAP towards COVID-19 infection prevention and its associated factors among pregnant women are crucial for policymakers, and stakeholders to design further strategies. Therefore, the main aim of this systematic review and meta-analysis was to estimate the pooled prevalence of pregnant women KAP towards COVID-19 infection prevention and associated factors in Ethiopia.

## Methods

### Study design and setting

Preferred Reporting Items for Systematic Review and Meta-Analysis (PRISMA) guideline was used for this review [23] (S1 Table). A systematic review and meta-analysis was conducted to estimate the pooled prevalence of pregnant women’s knowledge, attitude, and practice of COVID-19 infection prevention and its associated factors in Ethiopia.

### Search strategies and source of information

We have checked the PROSPERO database (http://www.library.ucsf.edu/) whether published or ongoing projects exist related to the topic to avoid any further duplication. Thus, the finding revealed that there were no ongoing or published articles in the area of this topic. Therefore, the protocol for this review is registered with ID = CRD42022314755. Comprehensive literature was searched until March 7, 2022 using international databases. Unpublished studies were sought from the official website of an international and/or local organization or university to retrieve related articles. The following search terms were formulated using PICO guidelines such as awareness, knowledge, perception, attitude, practice, COVID-19, coronavirus diseases, SARS CoV-2, pandemic, pregnant women’s. Medical Subject Headings (MeSH) and key terms had been developed using different Boolean operators ‘AND’ and ‘OR’ (S2 Table).

### Eligibility criteria

#### Inclusion criteria

1. Primary Studies (i.e. cross-sectional and case-control) reporting pregnant women’s knowledge, attitude, practice, and associated factors of COVID-19 infection prevention in Ethiopia were included in the current review.
2. All observational studies published in the English language only were included. It was due to financial constraints to translate the Amharic (local language) to the English language.
3. Pregnant women aged ≥18 years were included.
4. Both published and unpublished pre-print studies up to March 7, 2022, were included.

#### Exclusion criteria

1. Population other than pregnant women (i.e. general population, health care workers, high school students, college, and university students) were excluded.
2. Articles without full abstracts or texts and articles reported out of the outcome interest were excluded.
3. Citations without abstracts and/or full-text, commentaries, anonymous reports, letters, editorials, and reviews, were excluded after reviewing the full texts.
4. Studies conducted outside Ethiopia were excluded.
5. Studies published other than English language was excluded.

### Outcome measurements

The primary outcome was the pregnant women’s knowledge, attitude, and practice of COVID-19 infection prevention. Knowledge of pregnant women about COVID-19 was assessed using 15 items such as etiologic agent of COVID-19, clinical signs and symptoms of COVID-19, mode of transmission, and treatment options. Those who had scored greater than or equal to the mean value were coded as good knowledge [21, 24]. The attitude of pregnant women towards COVID-19 was assessed using 6 attitude-related items. Those who had scored greater than or equal to the mean value were coded as having positive attitude. Similarly, practice towards COVID-19 mitigation measures was assessed using 16 items that primarily focus on hand washing, maintaining physical distance, wearing a face mask, etc. Those who had scored greater than or equal to the mean value were coded as good practice. The secondary outcome was factors associated with pregnant women’s practice towards COVID-19 infection prevention measures including the socio-demographic variables (i.e. maternal age, residence, and level of education) [24, 25].

### Data extraction

All studies obtained from all databases were exported to Endnote version X8 software to remove duplicate studies. Then after, selected studies were exported to a Microsoft Excel spreadsheet. Two authors (AZ and TS) independently extracted all the important data using a standardized data extraction form which was adapted from the Joanna Briggs Institute (JBI) data extraction format [26]. For the first outcome (prevalence) the data extraction format included (the primary author name, year of publication, regions, sample size, and prevalence with 95% CI). Then after, we used 2 by 2 table format to extract data for the second outcome (associated factors of COVID-19 infection prevention practice). Finally, the log odds ratio for each factor was calculated using STATA version 14.0 software.

### Quality assessment

The modified Newcastle Ottawa Quality Assessment Scale (NOS) for cross-sectional studies was used to assess the quality of each study [27] (S3 Table). Two authors (AZ and TS) assessed the quality of each study including the quality of used methodology, sampling selection procedure, sample size, comparability and the outcome, and statistical analysis of the study. In the case of disagreement between two authors; the primary author (AM) was involved and discussed and resolved the disagreement. All included studies in systematic review and meta-analysis were cross-sectional.

### Data processing and analysis

All selected articles were entered into Microsocft Excel spreadsheet format and imported to STATA version 14.0 statistical software for analysis. The pooled prevalence of pregnant women’s knowledge, attitude, and practice towards COVID-19 was calculated using the weighted inverse variance random effect model. The Cochrane Q-test and I^2^ statistics were computed to assess heterogeneity among all studies. Accordingly, if the result of I^2^ was 0%, we considered it as no heterogeneity, 25% it was low heterogeneity, 50% would be medium heterogeneity, and 75% would be substantial heterogeneity [28]. Funnel plot and Eggers test were done to assess publication bias. The p value >0.05 indicated that there was no publication bias. Subgroup analysis was done based on the year of publication and study region. The pooled prevalence of pregnant women’s KAP towards COVID-19 with 95%CI was presented using a forest plot.

## Results

### Characteristics of included studies

**Fig 1:**
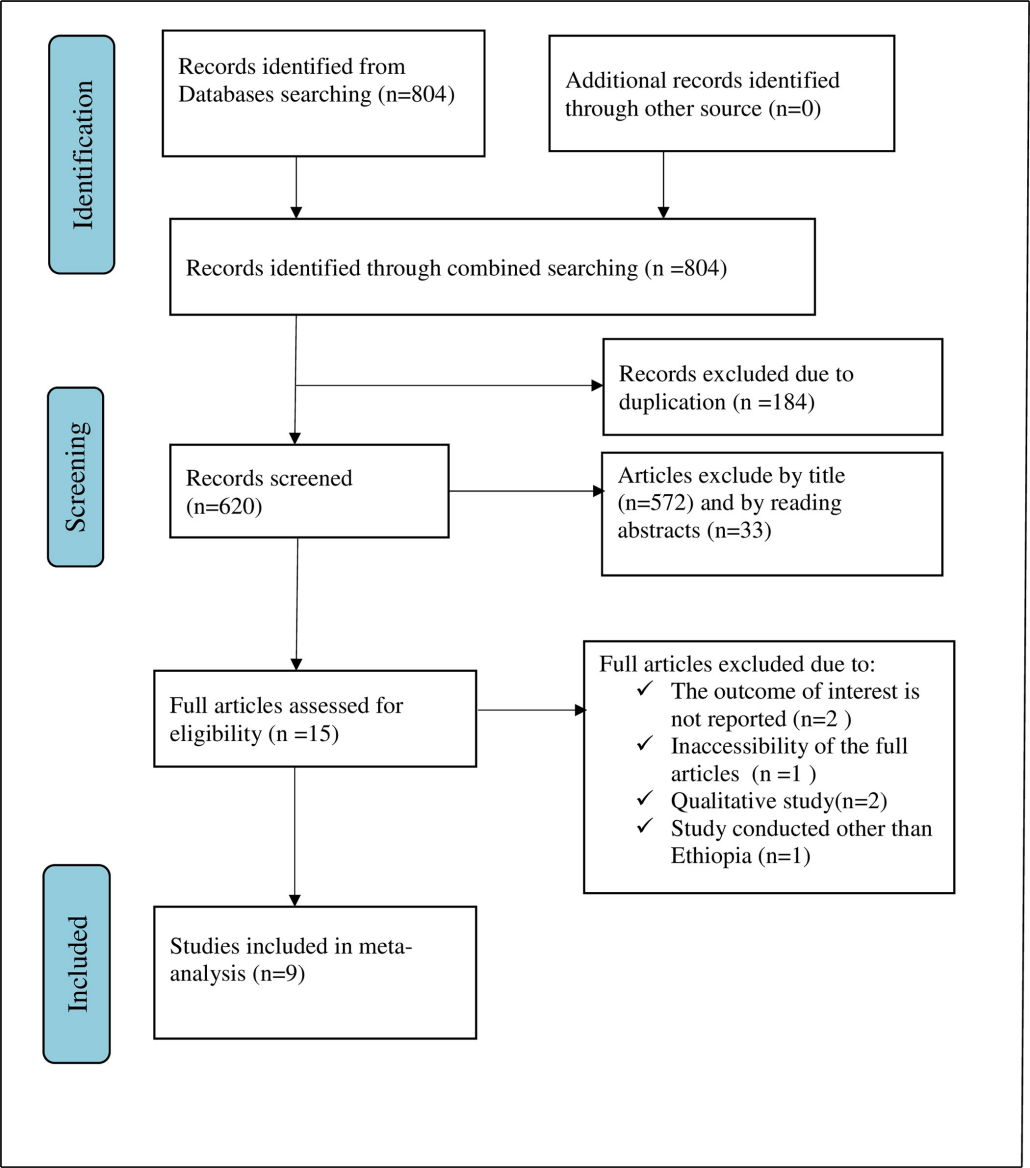
Flow chart of selection for systematic review and meta-analysis.

A total of 804 articles were retrieved using a search strategy about knowledge, attitude, practice towards COVID-19 infection prevention, and its associated factors among pregnant women in Ethiopia. International databases such as PubMed, Scopus, Google scholar, African Journals Online, and Web of Sciences were searched. Duplicates (184) were removed and 620 articles remained. After reviewing, (n = 572) articles were excluded by title, and (n = 33) articles were excluded by reading abstracts. Therefore, 15 full-text articles were accessed and assessed for inclusion criteria, resulting in the further exclusion of 6 articles primarily due to reasons such as inaccessibility of full articles, failure of reporting the outcome of interest, used qualitative study design, and studies that were conducted outside Ethiopia (Fig 1). Finally, 9 studies fulfilled the inclusion criteria to undergo the final systematic review and meta-analysis (Table 1).

**Table 1:** Characteristics of included studies in this systematic review and meta-analysis.

Authors	Study period	Publication year	Region	Sample size	Good knowledge (%)	Good attitude (%)	Good Practice (%)	Study design	Study setting	Study quality
Zeleke AM, & Bayeh GM [25]	June 1 to 30, 2021.	2022	Amhara	538	67.3	43.0	51.6	CS	Institutional based	7
Ayele AD. et al [29]	May 25 -June 15, 2020	2021	Amhara	405	46.8		47.6	CS	Community based	8
Belayneh M. et al [30]	March and April 2021	2022	Amhara	458	62.1	73.4	52.6	CS	Facility based	7
Besho M. et al [20]	July to August 2020	2021	Oromia	415	75.4	51.7	43.6	CS	Institutional based	8
Fikadu Y. et al [18]	July 27–August 27, 2020	2021	SNNP	403	54.8		76.2	CS	Institutional based	9
Temesgan WZ. et al [19]	July 1st to 30th, 2021	2022	Amhara	663	62.1		44.8	CS	Community	8
Degu A. et al [24]	June 05 to 26, 2020	2021	Amhara	403	52.1	52.6		CS	Institutional-based	
Silesh M. et al [22]	May 1 to 30, 2021	2021	Amhara	396	70.5	87.6	56.1	CS	Institutional based	9
W/Mariam T. et al [21]	July and August 2020.	2021	Amhara	422	55		47.4	CS	Institutional based	7

**NB.** SNNP; Southern Nation Nationalities People, CS: Cross-sectional

### Pregnant women’s knowledge, attitude, and practice COVID-19

**Fig 2:**
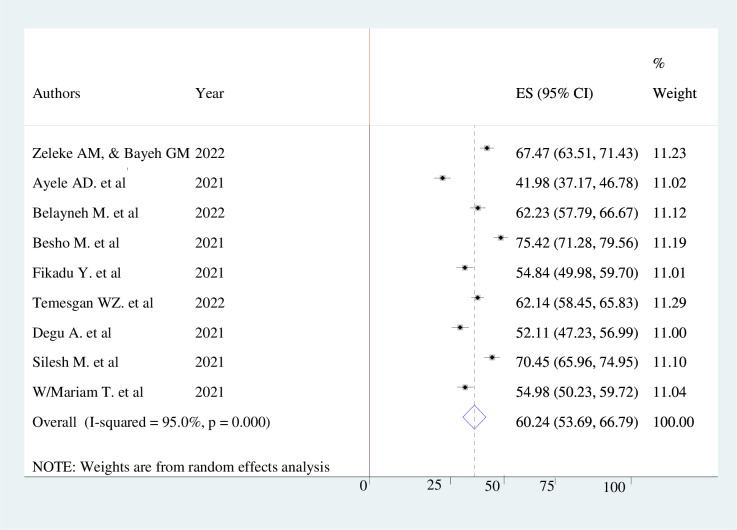
Pooled level of good knowledge of pregnant women’s towards COVID-19 infection prevention.

**Fig 3:**
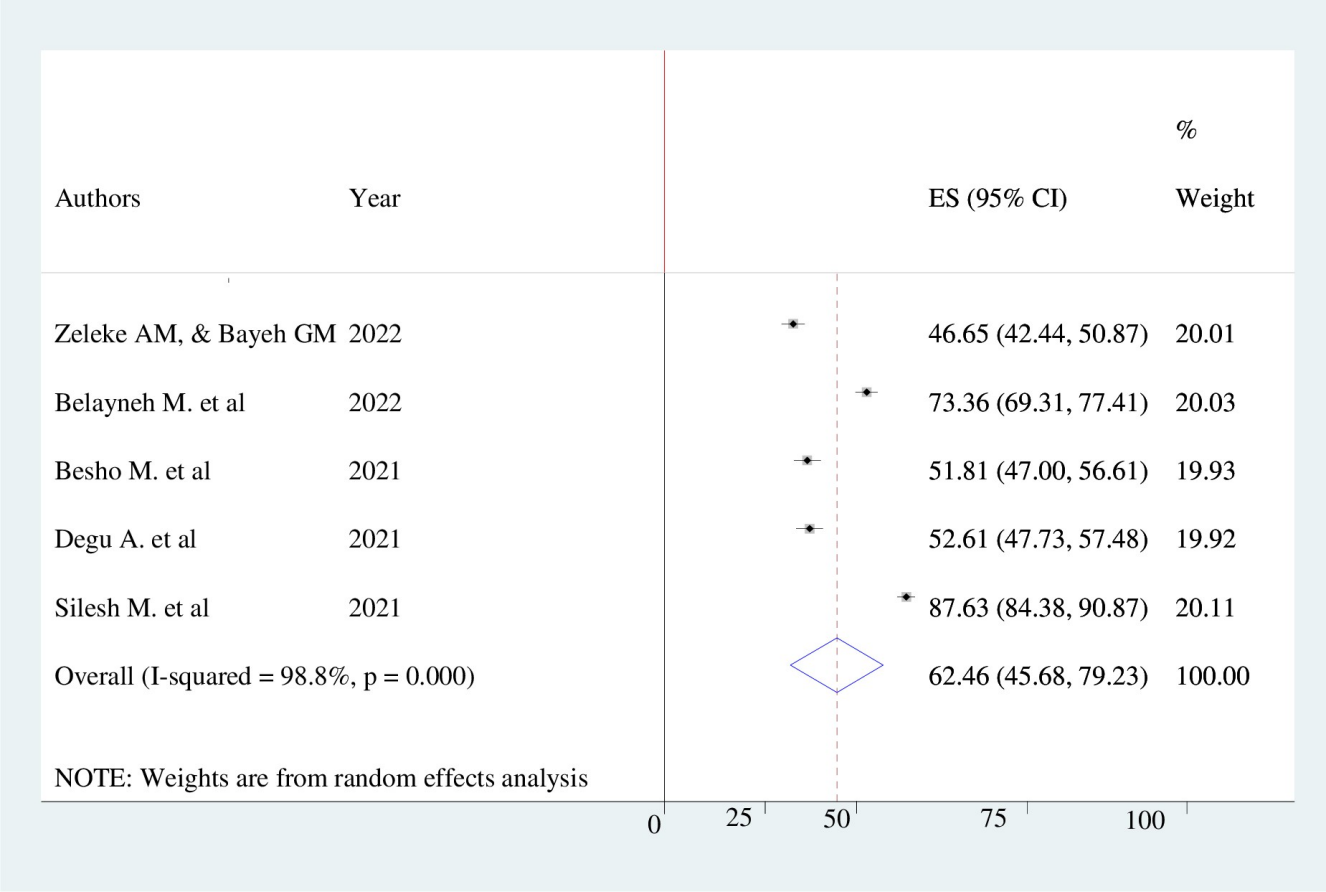
Pooled level of positive attitude of pregnant women’s towards COVID-19 infection prevention.

**Fig 4:**
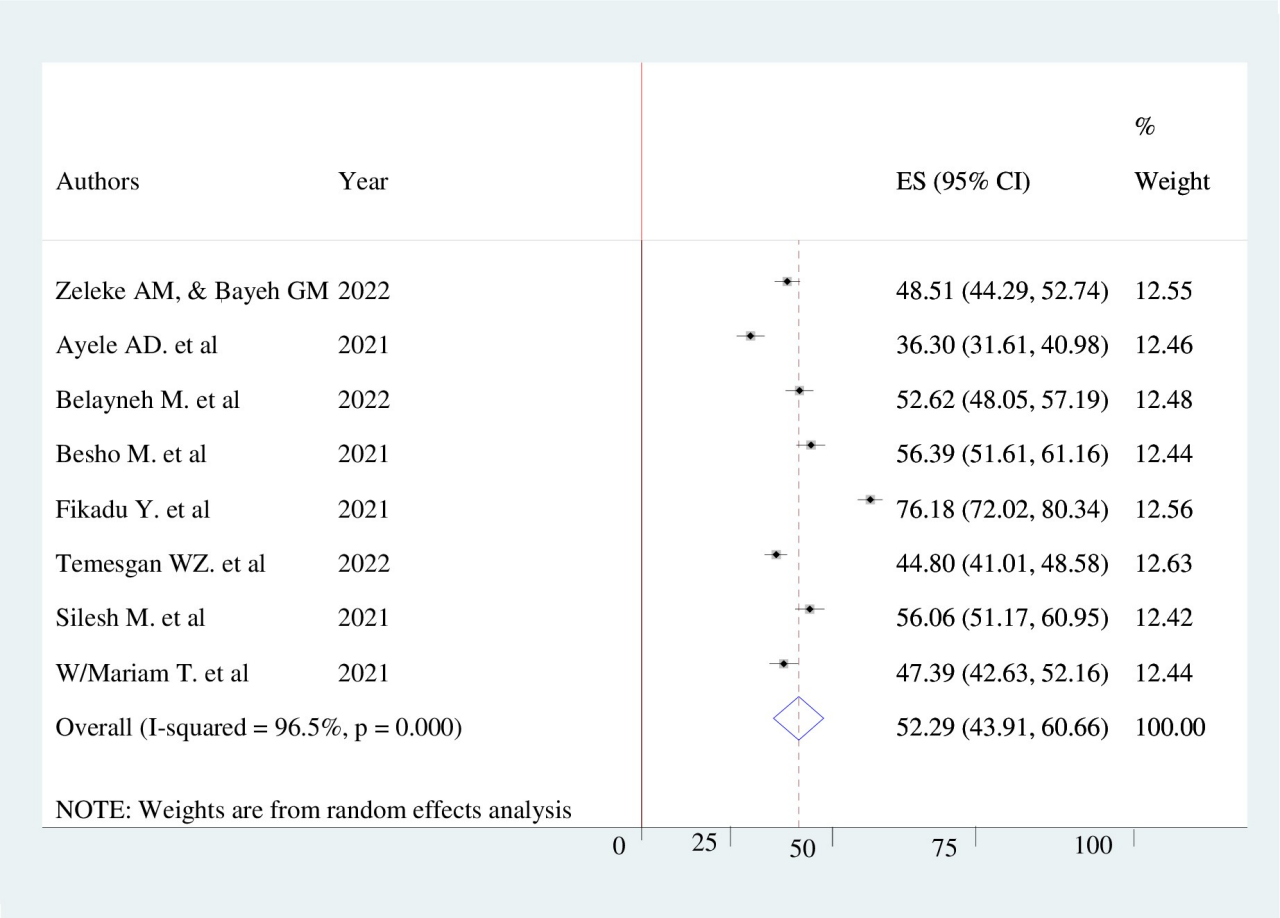
Pooled level of good practice of pregnant women’s towards COVID-19 infection prevention.

The pooled prevalence of knowledge, attitude, and practice towards COVID-19 infection prevention among pregnant mothers in Ethiopia were 60.24% (95% CI 53.69–66.79, *I*^*2*^ = 95%), 62.46% (95% CI; 45.68–79.23, *I*^*2*^ = 98.8%), and 52.29% (95% CI; 43.91%-60.66%, *I*^*2*^ = 96.5%) respectively. The finding was presented using a forest plot (Figs 2–4).

### Publication bias

**Fig 5:**
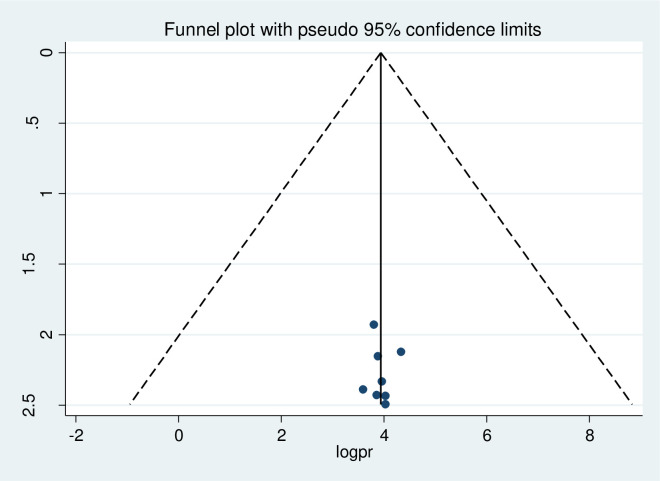
Assessment of publication bias for knowledge of pregnant women’s towards COVID-19 infection prevention.

**Fig 6:**
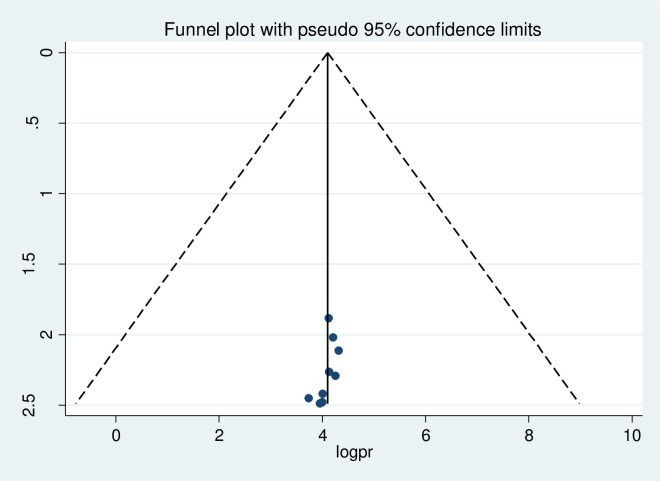
Assessment of publication bias for attitude of pregnant women’s towards COVID-19 infection prevention.

**Fig 7:**
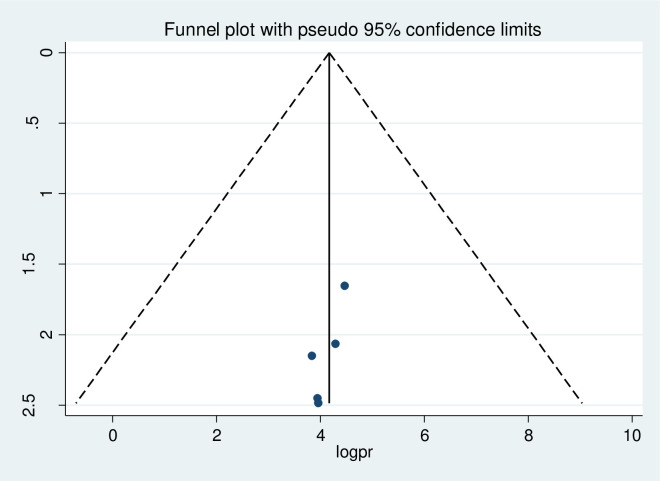
Assessment of publication bias for practice of pregnant women’s towards COVID-19 infection prevention.

A funnel plot was done at a significance level of less than 0.05. The Egger’s regression test was not statistically significant (p>0.05) confirming no evidence of publication bias, as presented by the funnel plot (Figs 5–7).

### Subgroup analysis by region

The finding of subgroup analysis by region showed that the level of good knowledge in the Oromia region and the SNNP region was 75.42% (95%CI; 71.28–79.56, *I*^*2*^ = 0.0%, *P* = 0.00) and 54.84% (95%CI; 49.98–59.70, *I*^*2*^ = 0.0%, P = 0.00) respectively. On the other hand, the pooled level of good knowledge in the Amhara region was 59.81% (95% CI; 58.16–61.46, I^2^ = 94.3%, *P* = 0.00). The prevalence of pregnant women’s practice towards COVID-19 infection prevention was 76.18% (95% CI; 72.02–80.34, *I*^*2*^ = 0.0%, *P* = 0.00) in the SNNP region. Similarly, the pooled prevalence of pregnant women’s practice towards COVID-19 infection prevention in the Amhara region was 47.35% (95% CI; 45.54–49.16, *I*^*2*^ = 87.7%, *P* = 0.00) (Table 2).

**Table 2:** Level of knowledge, attitude, and practice towards COVID-19 infection prevention among pregnant women in Ethiopia.

**Knowledge related articles**						
Variable	Characteristics	Pooled level of good knowledge		95%(CI)		I^2^(p-value)
By study region	Amhara	59.81		58.16–61.46		94.3%, (p = 0.00)
	Oromia	75.42		71.28–79.56		0.0%,(0.0)
	SNNP	54.84		49.98–59.70		0.0%,(0.0)
By year of publication	2021	59.51		57.62–61.40		96.6%, (p = 0.00)
	2022	63.98		61.67–66.28		56.0%, (p = 0.10)
**Attitude related articles**						
By study region	Characteristics	Pooled level of positive attitude	95%(CI)		I^2^(p-value)	
	Amhara	69.35	67.37–71.34		98.9%, p = 0.00)	
	Oromia	51.81	47.00–56.61		0.0%,(0.0)	
	SNNP					
By year of publication	2021	70.87	68.51–73.22		99.1%, (p = 0.00)	
	2022	60.55	57.63–63.47		98.8%, (p = 0.00)	
**Practice related articles**						
By study region	Characteristics	Pooled level of good practice	95%(CI)		I^2^(p-value)	
	Amhara	47.35	45.54–49.16		87.7%, (p = 0.00)	
	Oromia	56.39	51.61–61.16		0.0%,(0.0)	
	SNNP	76.18	72.02–80.34		0.0%,(0.0)	
By year of publication	2021	55.61	53.54–57.68		97.7%, (p = 0.00)	
	2022	48.15	45.75–50.55		70.2%, (p = 0.04)	

### Subgroup analysis by year of publication

The subgroup analysis by year of publication showed that by the end of 2021 the level of knowledge, attitude, and practice COVID-19 infection was 59.51%, (95%CI; 57.62–61.40, *I*^*2*^ = 96.6%, *P* = 0.00), 70.87%, (95%CI; 68.51–73.22, *I*^*2*^ = 99.1%, *P* = 0.00), and 54.49% (95%CI; 40.93, 68.04, *I*^*2*^ = 97.7%, *P* = 0.00) respectively. In the early 2022, the level of knowledge, attitude, and practice towards COVID-19 infection was 63.98% (95%CI; 61.67–66.28, *I*^*2*^ = 56.0%, *P* = 0.10), 60.55%, (95%CI; 57.63–63.47, *I*^*2*^ = 98.8%, *P* = 0.00), and 48.15% (95%CI; 45.75–50.55, *I*^*2*^ = 70.2%, *P* = 0.04) respectively (Table 2).

### Sensitivity analysis

A random effect model result showed that no single study has influenced the overall pooled prevalence of pregnant women’s knowledge, attitude, and practice towards COVID-19 infection prevention in Ethiopia (Table 3).

**Table 3:** Leave-out-one sensitivity analysis.

**Knowledge related articles**		
Study omitted	Pooled estimate (%)	95% Conf. Interval
Zeleke AM, & Bayeh G.	59.50	12.33–286.96
Ayele AD. et al	62.83	13.53–291.58
Belayneh M. et al	60.32	12.82–283.80
Besho M. et al	58.66	12.29–279.93
Fikadu Y. et al	61.12	13.19–283.20
Temesgan WZ. et al	60.23	12.25–296.06
Degu A. et al	61.43	13.26–284.44
Silesh M. et al	59.45	12.66–279.06
W/Mariam T. et al	61.14	13.14–284.31
**Attitude related variables**		
Study omitted	Point estimate (%)	95% Conf. Interval
Zeleke AM, & Bayeh GM	69.76	99.11–533.92
Belayneh M. et al	62.57	8.01–488.76
Besho M. et al	67.13	9.24–487.44
Degu A. et al	66.88	9.25–483.39
Silesh M. et al	56.05	6.07–516.91
**Practice related articles**		
Study omitted	Point estimate (%)	95% Conf. Interval
Zeleke AM, & Bayeh GM	51.77	79.56–280.08
Ayele AD. et al	53.60	10.15–282.91
Belayneh M. et al	51.13	9.63–271.31
Besho M. et al	50.72	9.64–266.72
Fikadu Y. et al	48.05	8.84–261.09
Temesgan WZ. et al	52.77	9.42–295.37
Silesh M. et al	50.79	9.71–265.80
W/Mariam T. et al	51.80	9.84–272.48

### Factors associated with COVID-19 infection prevention practice

In the meta-analysis; maternal age, residence, having secondary and above educational status, good knowledge, and fear of COVID-19 infection were factors associated with pregnant women’s practice towards COVID-19 infection prevention.

The odds of maternal age >25 years were 1.87 times more likely to have good preventive practices of COVID-19 infection than their counterparts (AOR = 1.87, 1.40–2.49). The odds of residing in an urban area were 2.23 times more likely to have good preventive practices for COVID-19 infection than those who had resided in rural areas (AOR = 2.23, 1.50–3.31). The odds of having secondary and above educational status were 3.36 times more likely to have good preventive practices for COVID-19 infection compared to having no formal education (AOR = 3.36, 2.46–4.58). The odds of having good knowledge about COVID-19 infection were 2.73 times more likely to have good preventive practices of COVID-19 infection than those who had poor knowledge (AOR = 2.73, 2.18–3.41). The odds of fear of COVID-19 infection were 2.60 times more likely to have good preventive practices of COVID-19 infection measures than their counterparts (AOR = 2.60, 1.78–3.80).

## Discussion

Immunologic, physiologic, and anatomical changes that occur during pregnancy cause suppression of the immune system, making pregnant women and their foetus more vulnerable to COVID-19 infection morbidity and mortality compared to non-pregnant women [7]. The current systematic review and meta-analysis was aimed to estimate the pooled prevalence of pregnant women KAP towards COVID-19 infection prevention and its associated factors in Ethiopia.

The overall knowledge of COVID-19 infection prevention among pregnant women was 60.24%. The finding is lower than studies conducted in India 90% [31], Saudi Arabia 78.3% [32], Egypt 75.4% [33], and Cameroon 84.19% [34]. The possible discrepancy might be due to differences among the study population and outcome variable measurements. For instance, a study conducted in Saud Arabia was among health care workers who had better awareness of the COVID-19 pandemic compared to pregnant mothers. Additionally, variation in outcome variable measurements might be the possible cause of discrepancy including median and percentages as a cut of point.

The level of attitude towards COVID-19 among pregnant mothers was found 62.46%, which is comparable with a study conducted in a low‐resource African setting 60.9% [35]. However, it is lower than the study conducted among the young Lebanese population 90% [36]. The discrepancy might be due to differences in study population and study variable measurement. For example, the study Lebanese was young and found mainly in the university which makes them have a positive attitude towards controlling the pandemic. Furthermore, the cut of points used to assess positive attitude among the Lebanese population was 3 out of 5 attitude-related questions, while in our study most of the included studies used median.

The pooled estimate of COVID-19 infection prevention practice among pregnant women was 52.29%. The finding is lower than studies conducted in South Africa 76% [17], India 92.7% [16], and China 89.7% [37]. The discrepancy might be due to the study period and study population difference. For instance, India is one of the countries which are severely affected by the pandemic compared to Ethiopia. Therefore, the government of India has been given a strong emphasis on COVID-19 infective prevention practice by social media and mass media platforms to enhance awareness of their community. However, the result is higher than a study conducted in Northern Ghana 46.6% [15]. The possible explanation might be differences in residence, access to the health care facility, study period, and outcome variable measurement. For example, a study conducted in Northern Ghana used Bloom’s cut of point.

Our study showed that maternal age was significantly associated with COVID-19 prevention practice. The finding is similar to previous studies conducted in n the Kingdom of Saudi Arabia and Mexico [38, 39]. This could be explained that in the older group of the population the fatality of COVID-19 might enhance compared to the younger population. Additionally, the older population might have age-associated comorbidities such as diabetes mellitus, heart diseases, and hypertension that might significantly compromise their immunity and in turn, makes them more likely adhere the COVID-19 mitigation measures. Thus, the World Health Organization prioritizes this group of the population for COVID-19 vaccination.

Pregnant women’s resided in urban areas were 2.23 times more likely to have good preventive practices of COVID-19 infection compared to those who had resided in rural areas. The finding is comparable with the literature review conducted globally [40]. The possible explanation might be those pregnant women who resided in urban areas have better access to health care facilities, television, and are able to read newspapers which might make them have aware of COVID-19 mitigation measures than their counterparts.

Pregnant women who had secondary and above educational status were 3.36 times more likely to have good preventive practices for COVID-19 infection than those who did not have formal education. The finding is similar to other studies conducted in the Democratic Republic of Congo [41] and Ethiopia [42]. The possible explanation might be those women who had secondary and above educational status has able to read newspapers, gather health information, and would have a better awareness of the WHO protocols of COVID-19 preventive measures.

Those pregnant women who had good knowledge of COVID-19 infection prevention measures were 2.73 times more likely to have good preventive practices for COVID-19 infection than those who had poor practice. The finding is similar to a study conducted in Oromia regional state, Ethiopia [43]. Moreover, this study revealed that pregnant women who had fear of COVID-19 infection were 2.6 times more likely to engage in COVID-19 infection prevention practice than those who did not fear COVID-19 infections. The finding is consistent with a scoping review conducted by Quadros S. et al [44]. The possible explanation might be the fact that COVID-19 causes significant loose of life which might create fear and make pregnant mothers to adhere COVID-19 mitigation measures.

### Strength and limitations of the study

To the best of the author’s knowledge, this systematic review and meta-analysis is the first study conducted in Ethiopia aimed to estimate the pooled prevalence of knowledge, attitude, and practice toward COVID-19 infection prevention among pregnant mothers in Ethiopia. Additionally, it explored factors associated with COVID-18 infection prevention practice. Therefore, it is relevant to inform maternal and child health policymakers and to upgrade the level of COVID-19 infection prevention practice. Web searches were restricted only to the English language, and the absence of studies in some regions of Ethiopia might make it unable to generalize the finding of this study.

## Conclusion

The pooled prevalence of pregnant women’s practice towards COVID-19 infection prevention measures was low. Maternal age, residence, secondary and above educational status, good knowledge, and fear of COVID-19 infection were factors associated with COVID-19 infection preventive practice. Therefore, policymakers, maternal and child health program planners, and stakeholders should target improving pregnant women’s awareness on COVID-19 prevention measures to control the pandemic.

## Supporting information

S1 Table PRISMA checklist. (DOCX)

S2 Table Search strategy for the databases. (DOCX)

S3 Table Newcastle-Ottawa quality assessment scale. (DOCX)

## Abbreviations

COVID-19: Coronavirus Disease 2019
SARS-COV-2: Severe Acute Respiratory Syndrome Coronavirus 2
WHO: World Health Organization
PRISMA: Preferred Reporting Items for Systematic Reviews and Meta-Analyses
