# Supplementary material for: Pregnant women’s knowledge, attitude, and practice towards COVID-19 infection prevention in Ethiopia: A systematic review and meta-analysis
Source: PLoS One. 2022 Oct 26;17(10):e0276692. doi: 10.1371/journal.pone.0276692 (PMC9605027; doi:10.1371/journal.pone.0276692)
Supplement: S2 Table — (DOCX) [file pone.0276692.s002.docx]

S2 Table: Search string for PubMed, Scopus, Google scholar, African Journals Online, and Web of Sciences.

| Databases | Search | Search terms |
| --- | --- | --- |
| PubMed | #1 | ‘‘Covid-19’’ OR ‘‘SARS Cov-2’’ OR ‘‘Coronavirus Diseases’’ |
|  | #2 | ‘‘Awareness OR ‘‘knowledge’’ |
|  | #3 | ‘‘Perception OR ‘‘attitude’’ |
|  | #4 | ‘‘Practice OR ‘‘Mitigation’’ |
|  | #5 | ‘‘Covid-19’’ OR ‘‘SARS Cov-2’’ OR ‘‘Coronavirus Diseases’’ AND ‘‘Awareness OR ‘‘knowledge’’ AND ‘‘Perception OR ‘‘Attitude’’ AND ‘‘Practice OR ‘‘Mitigation’’ |
|  | #6 | ‘‘Associated factors’’ OR ‘‘Determinant factors Or ‘‘Predictors’’ |
|  | #7 | ‘‘Pregnant women’s’’ OR ‘‘pregnant mothers’’ |
|  | #8 | ‘‘Covid-19’’ OR ‘‘SARS Cov-2’’ OR ‘‘Coronavirus Diseases’’ AND ‘‘Awareness OR ‘‘knowledge’’ AND ‘‘Perception OR ‘‘Attitude’’ AND ‘‘Practice OR ‘‘Mitigation’’ AND ‘‘Pregnant women’s’’ OR ‘‘pregnant mothers’’ AND ‘‘Associated factors’’ OR ‘‘Determinant factors Or ‘‘Predictors’’ |
|  | #9 | ‘‘Covid-19’’ OR ‘‘SARS Cov-2’’ OR ‘‘Coronavirus Diseases’’ AND ‘‘Awareness OR ‘‘knowledge’’ AND ‘‘Perception OR ‘‘Attitude’’ AND ‘‘Practice OR ‘‘Mitigation’’ AND ‘‘Pregnant women’s’’ OR ‘‘pregnant mothers’’ AND ‘‘Associated factors’’ OR ‘‘Determinant factors Or ‘‘Predictors’’ AND ‘‘Ethiopia’’ |
|  | #10 | Limit to March 7, 2022 |
|  | #11 | Only Humans |
|  | #12 | Only English language |
|  | #13 | ‘‘Covid-19’’ OR ‘‘SARS Cov-2’’ OR ‘‘Coronavirus Diseases’’ AND ‘‘Awareness OR ‘‘knowledge’’ AND ‘‘Perception OR ‘‘Attitude’’ AND ‘‘Practice OR ‘‘Mitigation’’ AND ‘‘Pregnant women’s’’ OR ‘‘pregnant mothers’’ AND ‘‘Associated factors’’ OR ‘‘Determinant factors Or ‘‘Predictors’’ AND ‘‘Ethiopia’’ AND Limit to March 7, 2022 AND Only Humans AND ‘Only English language’ |
| Scopus, Google scholar, African Journals Online, and Web of Sciences | #1 | ‘‘Covid-19’’ OR ‘‘SARS Cov-2’’ OR ‘‘Coronavirus Diseases’’ AND ‘‘Awareness OR ‘‘knowledge’’ AND ‘‘Perception OR ‘‘Attitude’’ AND ‘‘Practice OR ‘‘Mitigation’’ AND ‘‘Pregnant women’s’’ OR ‘‘pregnant mothers’’ AND ‘‘Associated factors’’ OR ‘‘Determinant factors Or ‘‘Predictors’’ AND ‘‘Ethiopia’’ |
