# Supplementary material for: Pregnant women’s knowledge, attitude, and practice towards COVID-19 infection prevention in Ethiopia: A systematic review and meta-analysis
Source: PLoS One. 2022 Oct 26;17(10):e0276692. doi: 10.1371/journal.pone.0276692 (PMC9605027; doi:10.1371/journal.pone.0276692)
Supplement: S3 Table — (DOCX) [file pone.0276692.s003.docx]

| **Newcastle-Ottawa Quality Assessment Scale scale for cross sectional studies** | **Selection** | | | | **Comparability** | **Outcome** | | **Total score** |
| --- | --- | --- | --- | --- | --- | --- | --- | --- |
|  | Representativeness (1) | Sample size  (1) | Non-respondents  (1) | Ascertainment of the exposure (risk factor)  (2) | The subjects in different outcome groups are comparable, based on the study design or analysis. confounding factors are controlled (2) | Assessment of the outcome  (2) | Statistical test  (1) |  |
| Shitie A. et al (15) | 1 | 1 | 1 | 1 | 1 | 1 | 1 | 7 |
| Tizazu MA. et al (14) | 1 | 1 | 1 | 1 | 1 | 2 | 1 | 7 |
| Cherie N.et al(18) | 1 | 1 | 1 | 1 | 1 | 2 | 1 | 8 |
| Asratie MH. et al(20) | 1 | 1 | 1 | 2 | 1 | 2 | 1 | 8 |
| Haile D. et al(17) | 1 | 1 | 1 | 1 | 1 | 2 | 1 | 7 |
| Atnafu A. et al(19) | 1 | 1 | 1 | 2 | 1 | 2 | 1 | 7 |
| Emiru AA. et al (10) | 1 | 1 | 1 | 2 | 1 | 2 | 1 | 8 |
| Sertsewold SG. Et al (16) | 1 | 1 | 1 | 1 | 1 | 2 | 1 | 7 |
| Tsega D. et al (25) | 1 | 1 | 1 | 1 | 1 | 1 | 1 | 7 |
